# Chitosan Nanoparticles Embedded in In Situ Gel for Nasal Delivery of Imipramine Hydrochloride: Short-Term Stage Development and Controlled Release Evaluation

**DOI:** 10.3390/polym16213062

**Published:** 2024-10-30

**Authors:** Samer Adwan, Teiba Obeidi, Faisal Al-Akayleh

**Affiliations:** 1 Department of Pharmaceutics and Pharmaceutical Technology, Faculty of Pharmacy, Zarqa University, Zarqa 13110, Jordan; 20199104@zu.edu.jo; 2 Department of Pharmaceutics and Pharmaceutical Technology, Faculty of Pharmaceutics and Medical Sciences, Petra University, Amman 11196, Jordan; falakayleh@uop.edu.jo

**Keywords:** chitosan, pluronic, nanoparticles, in situ gel, nasal drug delivery, imipramine

## Abstract

Imipramine hydrochloride (IMP), a tricyclic antidepressant used for major depression, enuresis, and neuropathic pain, is limited by gastrointestinal complications, low oral bioavailability (44%), and complex dosing requirements. This study aimed to explore a novel non-invasive nasal delivery system using chitosan nanoparticles (Cs NPs) embedded in an in situ gel to address the limitations of oral IMP administration. Cs NPs loaded with IMP were synthesized via ionic gelation and assessed for precision in drug concentration using a validated HPLC method. The particles were integrated into a thermoresponsive polymer, Pluronic F127, to form an in situ gel suitable for nasal administration. The formulation was characterized for gelation temperature, duration, viscosity, mucoadhesive strength, and overall gel robustness. Drug release kinetics and the controlled release mechanism were studied using ex vivo permeation tests with Franz diffusion cells and nasal sheep mucosa. The optimized nanoparticle formulation (F4-50) exhibited a consistent PS of 141.7 ± 2.2 nm, a zeta potential (ZP) of 16.79 ± 2.1 mV, and a high encapsulation efficiency of 67.71 ± 1.9%. The selected in situ gel formulation, F4-50-P1, demonstrated a gelation temperature of 33.6 ± 0.94 °C and a rapid gelation time of 48.1 ± 0.7 s. Transform-attenuated total reflectance infrared spectroscopy (ATR-IR) confirmed the compatibility and effective encapsulation of IMP within the formulation. The release profile of F4-50 included an initial burst release followed by a sustained release phase, with F4-50-P1 showing improved control over the burst release. The flux rates were 0.50 ± 0.01 mg/cm^2^/h for F4-50 and 0.33 ± 0.06 mg/cm^2^/h for F4-50-P1, indicating effective permeation. The developed chitosan nanoparticle-based in situ gel formulation provides a promising approach for the controlled release of IMP, enhancing therapeutic efficacy and patient compliance while mitigating the disadvantages associated with oral delivery.

## 1. Introduction

Nasal administration represents an innovative and advantageous route for drug delivery, distinguished by its convenience and non-invasive nature, which offers a significant advantage over more traditional methods. This approach, bypassing the gastrointestinal tract, not only avoids the potential for degradation of active pharmaceutical ingredients but also eliminates the discomfort and potential complications associated with parenteral injections [1]. The anatomical and physiological properties of the nasal cavity significantly contribute to its effectiveness as a drug delivery route. The nasal mucosa, characterized by high permeability, extensive vasculature, and a relatively low enzymatic activity, provides an optimal environment for the rapid absorption of drugs. Compared to other routes, this unique set of conditions ensures faster and more efficient absorption of medications, which is particularly beneficial for the delivery of time-sensitive therapeutics [2,3,4]. The nasal route effectively administers a diverse array of pharmacological compounds, ranging from small chemical entities to large biological macromolecules such as peptide/protein therapeutics and vaccines [5,6,7].

Cs NPs have become very useful as nanocarriers for drug, protein, and gene delivery systems. They have changed the way drugs are delivered without needles in many medical areas, such as brain diseases, cancer, and gastrointestinal and lung diseases. Benefiting from chitosan’s exceptional properties, such as excellent biodegradability, non-toxicity, biocompatibility, and bioadhesive nature, these nanoparticles offer unparalleled advantages in formulation design [8,9,10]. In contrast to other nanocarrier systems, Cs NPs are more stable, cause less harm, and are easy to make, which means they can be administered in a variety of ways. Furthermore, Cs NPs provide precise control over the release kinetics of active therapeutic agents, empowering tailored therapeutic interventions [11,12,13]. Cs NPs are synthesized using the bottom-up method, where molecules are assembled into defined structures in solution, and the top-down methods include milling, high-pressure homogenization, and sonication [14]. Small anionic molecules like phosphate, citrate, and sulfate induce Cs gelation, a process known as ionic gelation. In contrast, polyelectrolyte complexation is expected to occur when anionic macromolecules are used instead of small molecules [15]. Calvo et al. (1997) developed a mild adaptation of ionic gelation for preparing Cs NPs in a fully hydrophilic environment with sodium tripolyphosphate (STPP) as a crosslinker [16]. One significant challenge associated with chitosan nanoparticles is the “burst release” effect, where the rapid initial release of the drug may result in a reduced duration of drug activity, diminished therapeutic effectiveness, and an increased load on the liver and kidneys, aligning with previous observations [17]. To overcome this limitation, it is critical to develop a strategy that provides more precise control over the drug release kinetics. In response to this need, this study employs a thermoreversible in situ gel system for the incorporation of IMP-Cs NPs, aiming to enhance drug release management and address these concerns effectively.

Thermo-responsive systems are the most studied class of environment-sensitive polymer systems in drug delivery research. The term describes a system that reacts to temperature fluctuations, transitioning from a solution to a gel form within specific temperature ranges [18]. Poloxamer is a linear ABA-type triblock copolymer composed of hydrophilic end groups of PEO and hydrophobic core groups of PPO [(PEOa-PPOb-PEOc)n]. There are numerous types of poloxamers available with different properties, such as P127, P188, and P181, depending on the length and composition of the polymer chain. The underlying mechanism of gelation as a function of temperature suggests desolvation of the polymer accompanied by conformational changes of the side chains, leading to the displacement of the hydrating water molecules and modifications of the micelle orientation [19].

The current study used IMP as the model drug. With a molecular weight of 316.87 (g/mol), IMP exhibited a maximum plasma level at 2 to 8 h and has a plasma half-life ranging from 9 to 24 h. Consequently, frequent dosing is necessary to maintain therapeutic blood levels of the drug for long-term treatment. It serves as a second-line treatment for major depression and as an adjunctive therapy for nighttime wetting in children over the age of 6 years. It has other off-label uses, including chronic neuropathic pain (including diabetic neuropathy) and panic disorder [20]. The present work aims to formulate and evaluate a new and alternative strategy for the delivery of IMP via a non-invasive route of administration using IMP-Cs NPs ISG. This would reduce side effects, improve bioavailability, control drug release, and improve patient compliance.

## 2. Materials and Methods

### 2.1. Materials

Imipramine hydrochloride, Low molecular weight chitosan, Pluronic F-127, Sucrose, and Mannitol were purchased from Sigma-Aldrich^®^ (St. Louis, MO, USA). Sodium tripolyphosphate and Perchloric acid were purchased from Fischer Chemical (HK) ^®^ limited (Guangzhou, China), and Glacial acetic acid was purchased from Honeywell^®^ (Raunheim, Germany). Triethylamine was purchased from TEDIA^®^ (Tedia Way, Fairfield, OH, USA). Sodium perchlorate was purchased from Alpha chemika^®^ (Mumbai, India). Acetonitrile HPLC grade Sisco Research Laboratories Pvt Ltd. (Mumbai, India).

### 2.2. The Chromatographic Method

The IMP analysis was conducted using a JASCO LC-4000 Series HPLC system (Tsukuba, Japan) equipped with a binary pump, degasser, autosampler, and ultraviolet (UV) detector. Reversed-phase chromatography was employed for sample analysis, utilizing a Fortis^®^ ODS-C18 column (250 × 4.6 mm ID, 4 μm) RP-18. The flow rate was set at 1.5 mL/min, with UV detection at 250 nm. The autosampler temperature was maintained at 40 °C throughout the analysis. To prepare the mobile phase, 400 mL of Sodium perchlorate (NaClO_4_) was transferred to a 1000 mL volumetric flask, followed by the addition of 600 mL of acetonitrile (ACN) and 1 mL of triethylamine (TEA). The solution’s pH was adjusted to 2 using perchloric acid. Subsequently, the mobile phase was degassed by sonication for 60 min.

The method underwent validation according to ICH guidelines Q2(R1), 2005, covering parameters such as selectivity, system suitability, linearity, range, precision, intermediate precision, recovery, robustness, and limits of detection and quantification.

(1) $$\mathrm{LOD}=3.3\;\times \;\frac{SD}{S}$$

(2) $$\mathrm{LOQ}=10\;\times \;\frac{SD}{S}$$

### 2.3. Preparation of Cs NPs

Cs NPs were synthesized using low molecular weight Cs and STPP following a modified method [16] based on ionic crosslinking. Initially, Cs solutions of varying concentrations (0.5, 1, 1.5, 2, 2.5, and 3 mg/mL) were prepared by dissolving Cs in a 2% *v*/*v* acetic acid solution. The Cs solutions were stirred overnight at 200 rpm and room temperature to ensure complete mixing. Subsequently, the solutions were filtered through a 0.45 μm syringe filter to eliminate any impurities or undissolved Cs particles. Concurrently, STPP solutions of corresponding concentrations (0.5, 1, 1.5, 2, 2.5, and 3 mg/mL) were prepared by dissolving STPP in double distilled water. Following preparation, 20 mL of the STPP solution was added dropwise into 20 mL of Cs solution at a rate of 0.5 mL/min under constant magnetic stirring at 500 rpm and maintained at 25 °C. The resulting Cs NPs were isolated from excess STPP and other impurities using cellulose dialysis membranes (MWCO 12,000–14,000 KD). The purified Cs NPs were then mixed with 5% *w*/*v* sucrose as a cryoprotectant. The dispersion was gently shaken until the cryoprotectant fully dissolved, followed by freezing overnight in an ultra-low temperature deep freezer at −86 °C (Operon^®^, Incheo, Republic of Korea). After freezing, the dispersion was lyophilized using a lyophilizer (Labconco^®^, Fullerton, UK) at a pressure of 0.105 Torr and a temperature of −50 °C for 48 h. Following lyophilization, the Cs NPs were reconstituted by sonication for 10 min and manual shaking in double distilled water before further experimental use.

### 2.4. Preparation of IMP-Cs NPs

For the preparation of IMP-Cs NPs, IMP, at doses of 10 and 50 mg, was dissolved in Cs solution at a concentration of 2 mg/mL for 30 min prior to the addition of the STPP solution. The resulting IMP-Cs NPs were separated from the excess unloaded drug using cellulose dialysis membranes (MWCO 12,000–14,000 KD). The purified IMP-Cs NPs were then mixed with 5% *w*/*v* sucrose as a cryoprotectant. The dispersion was gently shaken until the cryoprotectant fully dissolved, followed by freezing overnight in an ultra-low temperature deep freezer at −86 °C (Operon^®^, Incheon, Republic of Korea). After freezing, the dispersion was lyophilized using a lyophilizer (Labconco^®^, Fullerton, UK) at a pressure of 0.105 Torr and a temperature of −50 °C for 48 h. Following lyophilization, the IMP-Cs NPs were reconstituted by sonication for 10 min and manual shaking in double distilled water before further experimental use.

### 2.5. Characterization of the Cs NPs and the IMP-Cs NPs

The mean PS and PDI were determined using a particle size analyzer (Brookhaven Instruments, New York, NY, USA), employing photon correlation spectroscopy. Each sample was diluted tenfold with filtered, double-distilled water using a 0.22 µm Millex syringe filter to prevent multi-scattering phenomena. Measurements were conducted using disposable sizing cuvettes at 25 °C, with each sample analyzed three times. Additionally, the ZP was measured using a Malvern Zetasizer (Malvern Panalytical Ltd., Malvern, UK), following the same procedure used for PS analysis but utilizing a clear disposable zeta cell.

### 2.6. Morphology Observation by Transmission Electron Microscope (TEM)

TEM imaging was performed to characterize polymeric micelles using a FEI Morgagni 268 microscope (FEI, Hillsboro, OH, USA) with an accelerating voltage of 100 kV. The samples were stained with 2% phosphotungstic acid, placed on a copper grid, and exposed under an infrared lamp for 10 min.

### 2.7. Calculation of the Percentage Encapsulation Efficiency and Percentage Loading Capacity

The prepared IMP-Cs NPs were isolated from the unloaded IMP using an indirect method involving a dialysis membrane. Samples were withdrawn from the dialysis medium and then analyzed using HPLC to determine the percentage encapsulation efficiency (%EE) (Equation (1)). The percentage loading capacity %LC represents the amount of drug loaded per unit weight of the nanoparticle (Equation (2)).

%EE = (total amount of IMP − free amount of IMP)/total amount of IMP (3)

%LC = (total amount of IMP − free amount of IMP)/total weight of NPs (4)

### 2.8. Preparation of Simulated Nasal Electrolyte Solution (SNE)

SNE was prepared by dissolving 8.77 (g/L) sodium chloride (NaCl), 2.98 g/L potassium chloride (KCl), and 0.59 g/L anhydrous calcium chloride (CaCl_2_) in double-distilled water to obtain a solution with a pH of 5.6 which mimics the physiological pH of the nasal mucosa (4.5 6.5) [21].

### 2.9. Formulation and Characterization of IMP-Cs NPs ISG

PF-127 was employed to formulate IMP-Cs NPs within a thermo-reversible gel using the cold method originally described by Schmolka in 1972 [22]. To prepare the polymer base, PF-127 was dissolved in cold water at concentrations ranging from 5 to 22% *w*/*v* at a temperature of 4 °C. A magnetic stirrer set at 100 rpm was used to ensure complete dissolution over 60 min, achieving a homogeneous dispersion. This solution was then allowed to swell and hydrate by storing it overnight at 6 °C in a refrigerator.

For the preparation of the IMP-Cs NPs ISG, a precisely weighed amount of the optimized lyophilized formulation (F4-50), equivalent to 50 mg of IMP, was reconstituted in half of the intended final volume of double-distilled water. The suspension was stirred at 50 rpm for 5 min, then gradually mixed with the selected PF-127 solutions (18 and 20% *w*/*v*) under gentle stirring at 50 rpm for an additional 10 min at 4 °C. The resulting dispersion was stored overnight at 6 °C to ensure a uniform distribution of IMP-Cs NPs within the PF-127 matrix, ultimately producing two distinct formulations: F4-50-P1 at 18% *w*/*v* and F4-50-P2 at 20% *w*/*v*. The prepared formulations were characterized in terms of various parameters, including appearance, pH, gelling temperature, gelling time, viscosity, mucoadhesive strength, gel capacity, and gel strength. All samples were measured in triplicate, and the results are expressed as the mean ± SD.

### 2.10. Gelation Temperature

The temperature at which the liquid phase transforms into a gel is defined as the gelling temperature [23]. It was determined using the test tube inversion method [24]. To determine the gelling temperature, 2 mL of the prepared formulation was transferred to a 10.0 mL test tube and immersed in a thermostatic water bath. The temperature of the water bath was gradually increased at a rate of 0.5 °C/min to specified temperatures in the range of 24–50 °C and left to equilibrate for 5 min at each new temperature. Observations of the in situ gel surface were made at each temperature point by tilting the test tube 90°, and the temperatures at which a stiffened gel formed and the surface remained immobile within 30 s were measured using an inserted thermometer. The gelation temperature was recorded in seconds.

### 2.11. Gelation Time

In situ, gels are characterized by their ability to gel at a specific temperature. The gel time must be adequate for the prescribed formulation [23]. The gelation time was determined using a procedure described by Rarokar et al. (2016). Briefly, 2 mL of the formulation was transferred to a test tube containing 2 mL of SNE and immersed in a thermostatic water bath at the measured gelation temperature [25]. The time required for all the liquid to completely gel was then observed, and the gelation time in seconds was recorded.

### 2.12. Viscosity

The viscosity was evaluated at 35 °C using a Brookfield viscometer (Funjilab^®^, New York, NY, USA) with spindle 62 at 50 rpm. The formulation was converted to a gel state with the help of a thermostatic water bath whose temperature was set at 35 °C, and the viscosity was measured in cps units.

### 2.13. Mucoadhesive Strength

The mucoadhesive strength is the force required to detach the formulation from nasal mucosal tissue [26]. A modified balance method was employed for the experiment. A sheep nasal mucosa was cut into 2 cm^2^ pieces and glued to a glass slide, ensuring the smooth surface of the nasal mucosa faced the upper side of the glass. The glued mucosa was then wetted with SNE (pH 5.6) by filling a beaker with SNE on the right-hand side of the balance. The setup was positioned under the right side of the pan. A thin film of the formulation was spread on the lower surface of the right pan. The right pan was lowered and spread with gel by removing the beaker from the left pan. Afterward, the pan was left for 2 min to ensure contact between the nasal mucosa and the gel. Then, using a burette, water was slowly added to the left pan until the nasal mucosa separated from the gel film. The mucoadhesive force was calculated by determining the weight required to separate the mucosa. The force was expressed in dyne/cm^2^ (Equation (3)):

*Mucoadhesive strength* = m × g/A (5)

where m is the weight required for detachment (g), g is the acceleration due to gravity (980 cm/s^2^), and a is the surface area of the nasal mucosa (cm^2^).

### 2.14. Gel Strength

The time required for a 20 g metallic ball to travel through the gel is indicative of its rigidity [26]. An accurately weighed quantity of 20 g of the formulation was placed in a 50.0 mL graduated cylinder and allowed to form a gel in a thermostatic water bath at 37 °C. Then, the metallic ball was placed on the gelled solution, and the time required for the weight to sink down (2 cm) through the gel was recorded [27].

### 2.15. ATR-FTIR

ATR-FTIR (PerkinElmer^®^, Llantrisant, UK) was employed to analyze the physical and chemical interaction between pure components and engineered formulations [28]. In this study, the possible interactions of IMP with Cs NPs formulation components were determined. All individual components, including polymers, the drug, their physical mixture, and developed NPs, were examined by an ATR-FTIR spectrometer. Physical mixtures were prepared by intimate trituration of equimolar quantities of 20 mg of IMP, Cs, and STPP, agitated by mortar for 10 min to obtain a homogeneous mixture. Samples were placed directly on the small crystal spot after cleaning with methanol, and then the arm was reversed and rotated down to push the sample onto the crystal phase for better contact.

### 2.16. In Vitro Release Study

The in vitro release of IMP from imipramine solution (IMP-solution), IMP-Cs NPs (F4-50), imipramine PF-127 solution (IMP-ISG), and IMP-Cs NPs ISG (F4-50-P1) was assessed using the dialysis technique, whereby a dialysis membrane (MWCO 12,000–14,000 kDa) was treated with SNE (pH = 5.6) overnight. Samples weighing equivalent to 50 mg of IMP were dispersed in 2 mL of SNE (pH 5.6) or PF-127 (18% *w*/*v*). After clamping the lower end of the membrane, samples were immersed in 20 mL SNE and transferred to tubes placed in a shaker incubator (JSR^®^, Gongju, Republic of Korea). The drug release was recorded at predetermined time intervals under sink conditions at 100 rpm at 35 °C over a period of 48 h. Samples of 1 mL from each tube were withdrawn from the release medium at specific time points (0.5, 1, 1.5, 2, 3, 4, 5, 6, 24, 30, and 48 h) and replaced with the same volume of fresh SNE (pH = 5.6).

### 2.17. Ex Vivo Permeation Study

To investigate the permeation efficiency of IMP-Cs NPs and IMP-Cs NPs ISG for the rapid transport of IMP across the nasal mucosa, ex vivo permeation studies were conducted using a Franz diffusion cell (PrepmeGear, Hellertown, PA, USA). These studies utilized sheep nasal mucosa sourced from a local slaughterhouse, following ethical sourcing standards. This approach ensures that the tissues used were obtained from animals already processed for meat, thereby minimizing additional ethical concerns and making efficient use of biological materials that would otherwise be discarded. On the day of the experiment, nasal tissue was treated with isopropyl alcohol to remove adherent mucus and fat. Then, it was cut to an appropriate size and mounted between the donor and receptor compartments of the Franz diffusion cell (volume of 12 mL and diffusing area of 1.767 cm^2^), with the mucosal side facing the donor compartment and the receptor compartment filled with SNE (pH = 5.6) and stirred magnetically at 100 rpm for proper mixing. The diffusion cell was set at 35 °C. IMP-Cs NPs (D1-50) (Equivalent to 50 mg IMP) were resuspended in 2 mL SNE (pH = 5.6) or PF-127 (18% *w*/*v*) and placed in the donor compartment. The area between the two compartments was wrapped with waterproof film to prevent evaporation; then, a clamp was used to firmly fix the dispenser compartment. Aliquots of 1 mL were collected from the receiver cell at predetermined time intervals (0.5, 1, 1.5, 2, 3, 4, 5, 6, 24, 30, and 48 h) and replaced immediately with the same volume of fresh media of SNE (pH = 5.6). The samples were filtered using a 0.45 µm membrane filter, and the amount of drug in the receptor media was analyzed using HPLC. The area value under the curve was converted into concentrations using a standard calibration curve. The cumulative amount of IMP permeated through the nasal mucosa per unit area (Q/A) was plotted versus time (t). The steady-state flux (J) (J, µg/cm^2^.h) was calculated from the slope of the linear portion of the (Q/A) versus (t) plot.

### 2.18. Statistical Analysis

*T*-test analysis or one-way analysis of variance was used to statistically analyze the results using GraphPad Prism software (ver. 6; GraphPad, Inc., San Diego, CA, USA). A statistically significant difference was defined as *p* < 0.05.

## 3. Results and Discussion

### 3.1. Optimization of Chromatographic Method

The USP 2021 method was slightly modified to reduce analysis time and chemical consumption. The optimized mobile phase used was NaClO4:ACN:TEA (400:600:1), pH 2 using perchloric acid. This method maintained selectivity, as evidenced by the absence of interfering peaks around the IMP peak. The retention time (RT) of the IMP peak was observed to be approximately 3.88 min instead of 11 min for the nonmodified USP 2021 method. Furthermore, the method demonstrated good separation, with a theoretical plate number (N value) of 5955 and a tailing factor (TF) of 0.93, meeting acceptance criteria (Center for Drug Evaluation and Research, 1994), where N > 2000 and TF < 2 are indicative of satisfactory performance. The optimized method underwent validation according to International Council for Harmonisation (ICH) guidelines Q2 (R1), 2005, encompassing assessments of selectivity, system suitability, linearity, range, precision, intermediate precision, recovery, robustness, limit of detection, and quantification. Table 1 presents a summary of the linear regression results for the calibration of IMP across a concentration range of 5 µg/mL to 75 µg/mL.

### 3.2. Fabrication and Characterization of Cs NPs and IMP-Cs NPs

The ionic gelation method was chosen for the preparation of Cs NPs in this study due to its simplicity, low toxicity, and scalability, which facilitate reproducible production in an aqueous medium without the need for organic solvents and high temperatures [29]. This method involves Cs, a cationic polysaccharide, undergoing gelation upon contact with STPP, forming electrostatic interactions between the amino groups of Cs and the negatively charged groups of STPP. Crosslinking agents enhance the physicochemical properties and stability of the polymer structure [30]. Cs-NPs are advantageous for controlling particle size, surface properties, and drug release for site-specific action [30]. LMW Cs was selected for its superior solubility, biocompatibility, bioactivity, biodegradability, and reduced toxicity. NPs smaller than 200 nm can be transported to the brain via endocytic pathways [31].

The impact of varying concentrations of Cs and STPP on the physicochemical properties of NPs was rigorously assessed to optimize the formulation parameters for the synthesis of Cs NPs. By systematically adjusting the concentrations of Cs and STPP, we identified the optimal ratios essential for fabricating Cs NPs with the desired nanometer-scale diameter and stability characteristics. An increase in the concentration of Cs was found to enhance nanoparticle stability, which was reflected in achieving an optimal particle size and improved opalescence. This effect is attributed to the increased intermolecular hydrogen bonding and electrostatic repulsion among the molecules. Selected formulations are presented in Table 2, where a Cs to STPP ratio of 2:1 was identified as the most effective. This ratio produced nanoparticles with optimal characteristics for nasal delivery, specifically designed for targeting the brain.

Based on data obtained in Table 2, F4 and F7 were selected for drug loading at two different concentrations of 10 and 50 mg. Across all formulations, particle size (PS) increased significantly with higher drug amounts (10 to 50 mg, *p* < 0.005), likely due to reduced Cs/STPP interaction as drug molecules compete with STPP [32]. This increase in drug amount also slightly decreased the PDI, indicating more uniform particles [33]. Additionally, ZP decreased slightly as the drug amount increased due to electrostatic interactions between protonated NH3+ of Cs and the drug, reducing surface charge [34,35]. All formulations maintained a positive charge, indicating optimal Cs concentration and unneutralized amino groups. Positive ZP enhances adhesion and transport properties due to electrostatic attraction with negatively charged cell membranes [32,36]. The %EE and %LC of the drug increased with higher amounts of IMP (10 to 50 mg) in each formulation (Table 3). This increase is likely due to electrostatic interactions between amino groups of chitosan and anionic parts of IMP. Moreover, hydrogen bonds could form between the hydroxyl groups of chitosan and the polar groups of IMP, contributing to the physical stability of their complex. The relatively low %LC can be attributed to the high water solubility and small molecular weight of IMP, which favors its partitioning into the hydrophilic phase rather than entrapment within Cs NPs [37].

**Figure 1:**
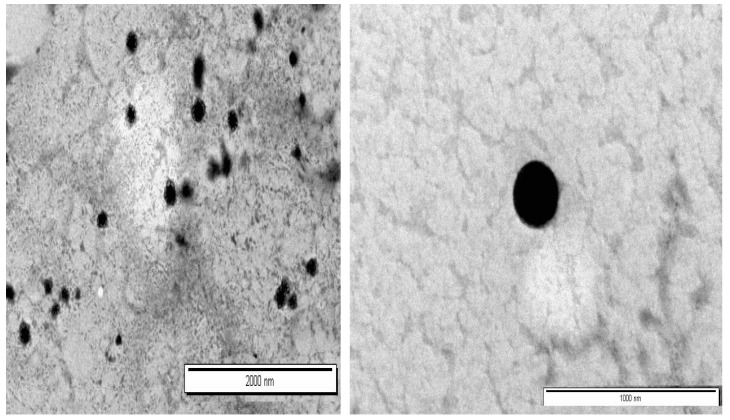
TEM images of Formulation F4.

The morphology of Formulation F4 was characterized using TEM, as illustrated in Figure 1. The images revealed that the nanoparticles exhibit a spherical shape. Spherical NPs are often preferred due to their higher packing density and uniform distribution in suspension. These characteristics are crucial as they facilitate more consistent drug release profiles, ensuring uniform delivery of the therapeutic agent. The incorporation of sodium tripolyphosphate (STPP) in the NP synthesis introduces some degree of surface roughness. This roughness results from the formation of cross-links within the NP structure, which is essential for stabilizing the particle and controlling the release of the encapsulated drug. The specific surface characteristics, including roughness induced by STPP, could potentially enhance the mucoadhesive properties of the nanoparticles or affect their cellular uptake, which are important parameters in the development of effective nanoparticle-based drug delivery systems [11,13].

### 3.3. Preparation and Characterization of IMP-Cs NPs ISG

Thermo-reversible polymers exhibit fluidity at low temperatures but undergo gelation when heated. Formulations based on thermo-sensitive polymers are engineered to prolong the retention of formulations within the nasal cavity, thereby enhancing drug absorption [26]. The polymer selection was based on its gelation properties at various concentrations of aqueous polymer solutions at nasal temperature (34 °C). PF-127, composed of a tri-block polymer network (PEO-PPO-PEO), was chosen due to its reverse thermal gelation behavior. In aqueous solutions at low temperatures, hydration layers form around PF-127 molecules through hydrogen bonding with water molecules. At higher temperatures, desolvation of the hydrophilic chains of the copolymer occurs, promoting hydrophobic interactions among the polyoxypropylene domains and resulting in gel formation [26]. Hence, PF-127 was selected for the incorporation of IMP-Cs NPs, preferably at low concentration levels, and exhibited desired properties for further studies.

The concentration of PF-127 required for preparation was determined through a preliminary study spanning a range of 5–22% *w*/*v* to ascertain the lowest concentrations exhibiting thermo-reversible properties below 34 °C (the physiological temperature of the nasal cavity). The concentrations selected based on gelation temperature below nasal temperature were 18% and 20% *w*/*v* PF-127. The pH of formulations F4-50-P1 and F4-50-P2 was found to be 5.4 and 5.5, respectively, ensuring compatibility with the nasal mucosa and minimizing irritation. The gelation temperature of F4-50-P1 and F4-50-P2 formulations was 33.6 °C and 30.3 °C, respectively, with gelation times of 48.1 and 44.5 s, respectively. These results are deemed ideal considering the physiological temperature of the nasal mucosa and mucociliary clearance time (t1/2 = 21 min), which dictate the temperature range and time limits for the sol-gel transition. Ideally, formulations should gel at the nasal temperature of 34 °C, and the gelation times indicate that the formulations undergo gel formation well below the mucociliary clearance time.

Moreover, the rheological behavior of the formulations was assessed, and viscosity increased with the concentration of PF-127 (1206 to 1443 cps). All formulations remained liquid at room temperature, transitioning to gel-like behavior with increased temperature, indicative of the change from Newtonian to non-Newtonian behavior. This viscosity change is attributed to a sudden increase in micellar concentration at higher temperatures [38]. Adequate mucoadhesive strength was observed for all formulations (Table 4), facilitated by the presence of polyoxyethylene groups in PF-127, which engage in hydrogen bonding with mucosal components. Although free polyoxyethylene groups may be less available for hydrogen bonding due to increased cross-linkage between polymer chains in thermo-reversible gel formulations, the presence of Cs NPs in this study obviated the need for additional mucoadhesive polymers. Previous research indicated that Cs, as a mucoadhesive polymer, interacted strongly with mucin through hydrogen bonding and electrostatic interactions between the amine function of Cs and sialic acid of mucin [39]. Gel strength measurements for F4-50-P1 and F4-50-P2 formulations presented in Table 4 are considered ideal, as gel strength values between 25 and 50 s are deemed adequate. Gel strengths of less than 25 s may result in rapid erosion and loss of integrity, while those exceeding 50 s may cause discomfort to the nasal mucosa [40].

### 3.4. ATR-FTIR Spectroscopy

**Figure 2:**
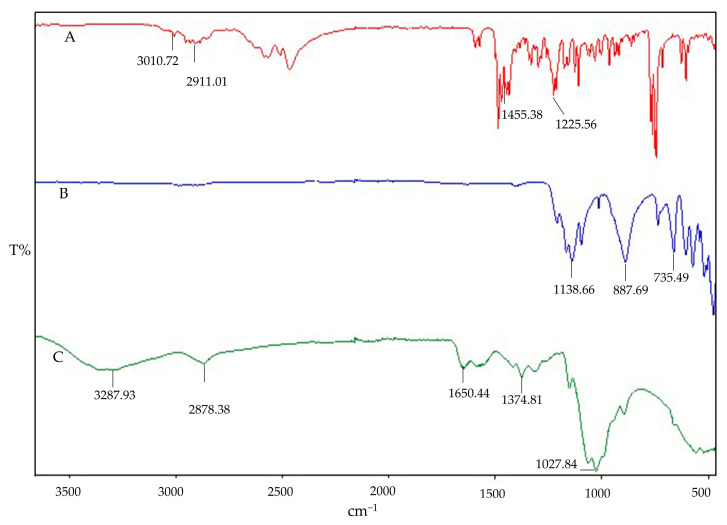
FTIR fingerprint of individual components: IMP (**A**), STPP (**B**), and (**C**) Cs.

**Figure 3:**
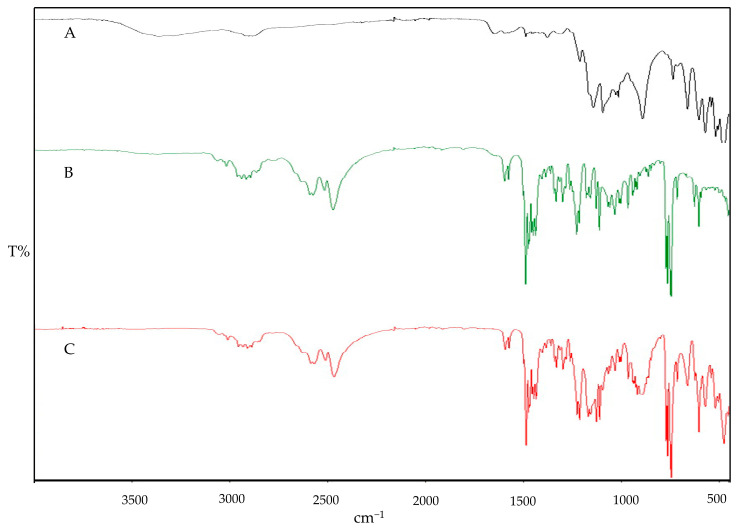
FTIR fingerprint of physical blends: Cs and STPP (**A**), Cs and IMP (**B**), and IMP and STPP (**C**).

The impact of interaction can also be qualitatively determined by FT-IR spectroscopy. The FTIR fingerprints of the pure components Cs, STPP, and IMP are presented in Figure 2. The Cs fingerprints exhibit the characteristic peak overlap of N–H and O–H stretching at 3287.93 cm^−1^, with an absorption band at 2878.38 cm^−1^, indicating asymmetrical stretching vibrations of C–H. The presence of N-acetylated groups of Cs is confirmed by the C=O stretching of amide I at 1650.44 cm^−1^ and amide II at 1582.36 cm^−1^. The wave numbers at 1374.81 cm^−1^ and 1027.84 cm^−1^ ensure the presence of CH3 (asymmetrical deformation) and C–O stretching, respectively. Similar characteristic peaks of pure Cs have been reported in previous studies [28]. The IR spectral analysis of STPP reveals strong P=O stretching vibrations at 1138.66 cm^−1^ and P–O stretching at 887.69 cm^−1^ and 735.49 cm^−1^, with characteristic peaks also reported elsewhere [41]. The IR spectra of IMP demonstrate C–H bond stretching in the frequency band region of 3010.72 to 2911.01 cm^−1^ confirming the presence of alkane methyl groups. Sharp peaks of C–H bending are observed at three different frequencies (1455.38, 1472.29, and 1485.88 cm^−1^), along with C–N bond stretching at two different frequencies: one at 1212.55 cm^−1^ and the second one at 1225.56 cm^−1^. Similar characteristic peaks have been observed in previous studies [42]. Possible interactions in the physical mixtures of IMP, Cs, and STPP have been examined and are shown in Figure 3. The binary blends (IMP and Cs), (IMP and STPP), and (Cs and STPP) indicate identical peaks with imperceptible shifts, indicating the absence of interaction between functional groups.

**Figure 4:**
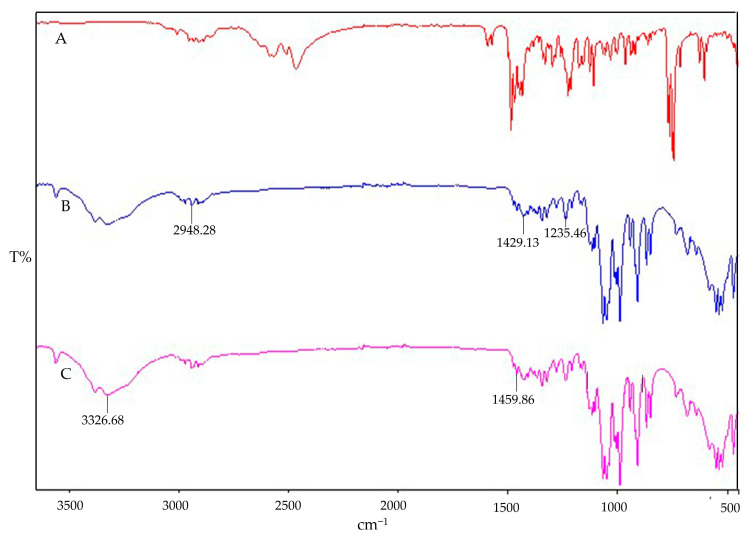
FTIR of IMP (**A**), Cs NPs (**B**), and IMP-Cs NPs (**C**).

Cs NPs displayed a broad absorption peak at 3326.68 cm^−1^ corresponding to O–H groups, indicating enhanced hydrogen bonding. Additionally, a significant shift in the amide II band from 1374.36 cm^−1^ to 1459.86 cm^−1^ was observed, which is indicative of Cs-STPP conjugation and interaction, as detailed in Figure 4. The absorption peaks of IMP-Cs NPs were found to decrease in intensity, suggesting interactions and entrapment of IMP within the Cs NPs. Specifically, the characteristic C–H bond stretching peaks of IMP shifted from 2948.28 cm^−1^ to 1429.13 cm^−1^, confirming the drug’s encapsulation. IMP also exhibited additional strong absorption bands at 1235.46 cm^−1^, which coincide with the chitosan bands, supporting the presence of IMP within the nanoparticle matrix. These spectral changes align with previous findings [28].

**Figure 5:**
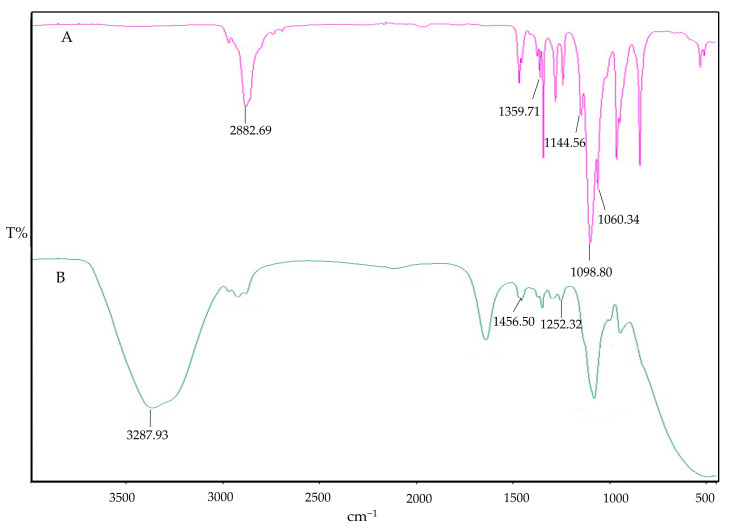
FTIR of PF-127 (**A**) and IMP-Cs NPs ISG (**B**).

The IR spectrum of PF-127 is characterized by its principal absorption peaks at 2882.69 cm^−1^ for aliphatic C–H stretch, 1359.71 cm^−1^ for in-plane OH bend, 1144.56 cm^−1^ for C-O stretch, 1098.80 cm^−1^ for C–N, and 1060.34 cm^−1^ for C–O–C ether linkages, as shown in Figure 5. In the context of IMP-Cs NPs ISG, there was a noted decrease in the strength of absorption peaks, indicating the interaction and effective entrapment of IMP. The characteristic C–H stretching peaks of IMP were adjusted slightly from 1455.38 cm^−1^ in free state to 1456.50 cm^−1^, evidencing the integration of IMP-Cs NPs into the PF-127 ISG system. Furthermore, IMP displayed additional strong absorption bands at 1252.32 cm^−1^, which overlapped with PF-127 absorption bands, as presented in Figure 5.

### 3.5. In Vitro Drug Release of IMP-Cs NPs

**Figure 6:**
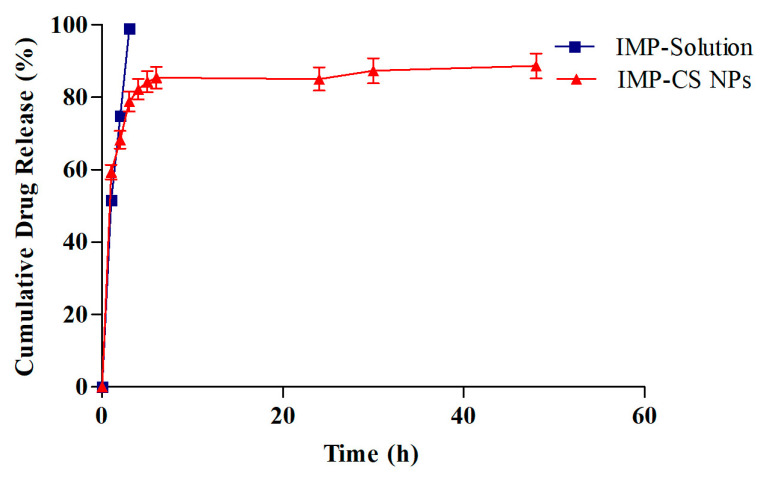
The in vitro drug release results. Plotted in terms of % released of IMP in SNF vs. time for IMP-Cs NPs (F4-50) and IMP-solution (Mean ± SD, *n* = 3).

Formulation F4-50 exhibited the highest %EE and %LC (Table 3). Drug release profiles for IMP from F4-50 and an IMP solution were studied in SNF (pH 5.6) at 35 °C for 48 h using a dialysis membrane and shaking incubator. The findings are graphically presented in Figure 6. Initially, a rapid release of IMP was observed within the first hour, with a cumulative release percentage reaching 55.82%. Subsequently, the release rate significantly decelerated, culminating in a controlled release profile of 88.63% by the end of the 48-h period.

The observed initial burst release is attributed to the loosely bound IMP molecules on the surface of the nanoparticles, which readily diffuse through the nanoparticle pores. This burst is predominantly driven by diffusion forces, where exposure to the aqueous environment facilitates the formation of hydrogen bonds at the superficial regions of the nanoparticles, enhancing the diffusion of the encapsulated IMP [33]. In the subsequent phase, the release mechanism transitions to diffusion through a gel-like layer on the surface of the nanoparticles. The Cs NPs undergo swelling and release the drug into the SNF through a diffusion process upon exposure to the liquid medium. This slower release phase is influenced by the rigid and hydrophobic core of the Cs NPs [17].

To quantitatively describe the drug release kinetics, the data were analyzed using various kinetic models, including First order, Zero order, Higuchi, and Korsmeyer-Peppas models. The regression coefficients (R^2^ values) obtained were 0.6685, 0.914, 0.9973, and 0.9711 for the First-order, Zero-order, Higuchi, and Korsmeyer-Peppas models, respectively, with the Higuchi model showing the highest level of fit. This model suggests that drug release from the F4-50 formulation is primarily governed by diffusion mechanisms. Further analysis using Labplot2 software (Version 2.0.8) confirmed the fit, with the Korsmeyer-Peppas model yielding a release exponent (n) of 0.5, indicative of a Fickian diffusion mechanism; this denotes a release that is dependent both on the diffusion across the polymer and subsequent relaxation of the polymer matrix [43].

The initial burst release can reduce drug efficacy and increase liver and kidney burden [17]. To mitigate this, a thermo-reversible in situ gel system was selected to incorporate IMP-Cs NPs, offering better control over drug release.

### 3.6. In Vitro Release Study of IMP-Cs NPs ISG

**Figure 7:**
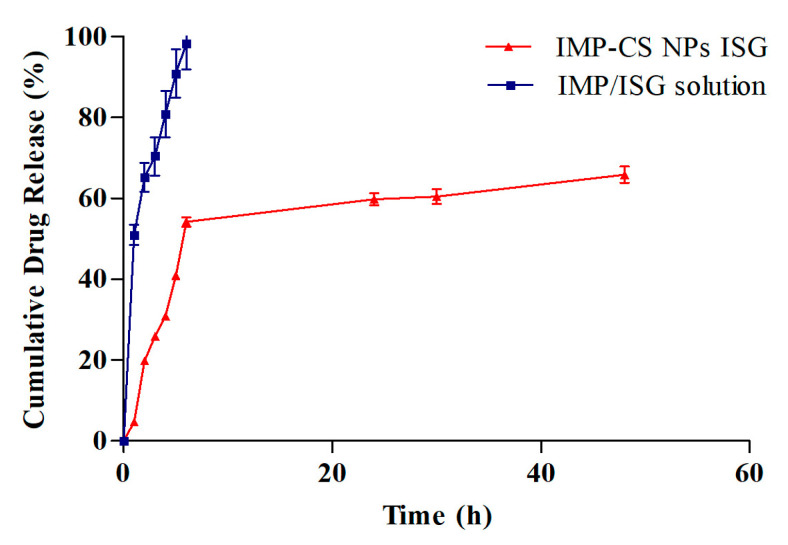
The in vitro drug release results. Plotted in terms of % released of IMP in SNF vs. time for IMP-Cs NPs ISG (F4-50-P1) and IMP/ISG solution (Mean ± SD, *n* = 3).

The in vitro drug release profiles for formulation F4-50-P1 and the IMP-ISG solution were assessed using a dialysis membrane setup, with results illustrated in Figure 7. Formulation F4-50-P1 demonstrated cumulative drug releases of 4.68%, 59.77%, and 65.89% at 1, 24, and 48 h, respectively. Notably, the incorporation of Pluronic F-127 (PF-127) in F4-50-P1 was observed to decelerate the drug release rate in comparison to the IMP-Cs NPs formulation. This slowing effect can be attributed to the increased viscosity induced by PF-127 in the gel matrix, which extends the diffusional pathways and potentially reduces the number and size of water channels within the gel structure, thus enlarging the micelles incorporated in the gel [38].

The release constants were computed from the slopes of the fitted plots, and the regression coefficients (R^2^ values) were derived for various kinetic models, including zero order, first order, Higuchi, and Korsmeyer-Peppas, with values of 0.858, 0.80, 0.9883, and 0.9422, respectively. Each model was meticulously chosen to shed light on various dimensions of the drug release process, providing a comprehensive view of how the drug interacts with the delivery matrix. The zero-order kinetics model, which assumes a constant drug release rate irrespective of the drug concentration, is particularly relevant for devices designed to deliver a uniform rate of drug release. However, it achieved an R^2^ value of 0.858, indicating that it might oversimplify the release dynamics, especially in systems where the release rate is not constant but varies with changes in the matrix and drug concentration. On the other hand, the first-order kinetics model, which posits that the release rate is proportional to the remaining drug concentration, proved to be moderately fitting with an R^2^ of 0.80. This model is generally suitable for systems where the release mechanism depends on the concentration gradient. Yet, our results suggest that it alone could not fully capture the complexities of drug release influenced by both matrix properties and the drug concentration gradient. The Higuchi model, which provided the highest compatibility with our data (R^2^ = 0.9883), bases its assumptions on Fickian diffusion, ideally suited for semi-solid or solid matrices where diffusion is the primary mechanism of drug release. Despite its robust application to our data, the model’s limitation is its exclusive focus on diffusion, not considering other influential factors such as polymer erosion or swelling of the matrix. The Korsmeyer-Peppas model, with an R^2^ of 0.9422, provided an excellent description of the drug release from our polymeric system, incorporating both diffusion and erosion processes. This alignment is consistent with prior research that has demonstrated similar release behaviors in polymeric drug delivery systems [23]. The detailed kinetic analysis underscores the significant impact of PF-127 on modifying drug release behaviors, emphasizing its role in enhancing the controlled release capabilities of the formulation.

### 3.7. Ex Vivo Permeation Study of IMP-Cs NPs and IMP-Cs NPs ISG

**Figure 8:**
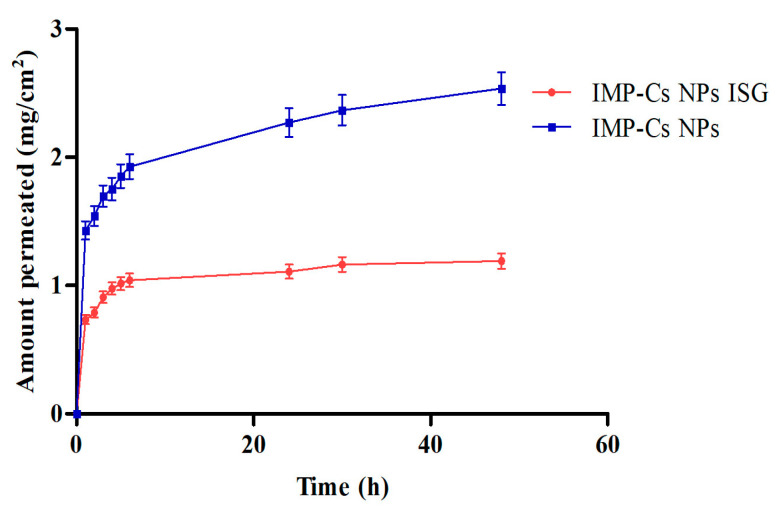
Ex vivo permeation study results of F4-50 and F4-50-P1 through nasal mucosa tissue using Franz cell (Mean ± SD, *n* = 3).

Ex vivo permeation studies through sheep nasal mucosa were conducted for IMP-Cs NPs and IMP-Cs NPs ISG (Figure 8). The permeation of IMP was quantified by plotting the cumulative amount permeated per area (mg/cm^2^) against time (h). The steady-state flux (J, µg/cm^2^.h) was obtained from the slope using linear regression. The flux rates were determined to be 0.50 ± 0.01 and 0.33 ± 0.06 mg/cm^2^/h for F4-50 and F4-50-P1, respectively. The interaction of the positively charged amino group of chitosan with the negatively charged sites on the cell membranes and tight junctions of the mucosal epithelial cells allows for the opening of tight junctions. IMP is a basic drug with a pKa of 9.4; thus, at the pH of the nasal mucosa (5.5–6.5), much of the drug remains in its protonated form and is transported through the opened tight junctions. Therefore, the permeation through the nasal mucosa is primarily attributed to paracellular transport through tight junctions [40]. The small PS of the optimized IMP-Cs NPs formulation and efficient dispersion of IMP in the matrix led to enhanced drug permeation from IMP-Cs NPs across the nasal mucosal layer, while the IMP-Cs NPs ISG formulation was able to control and extend the drug permeation. Similar findings have been reported previously [23].

## 4. Conclusions

This study focused on the development of IMP-Cs NPs ISG as a promising alternative strategy for delivering IMP to the brain. Various Cs NPs formulations were designed to accommodate two dose strengths of IMP (10 and 50 mg) and meticulously characterized for several parameters, including PS, PDI, ZP, ATR-FTIR, %EE, %LC, in vitro drug release, and ex vivo drug permeation. The selected formulation (F4-50) exhibited desirable characteristics such as a PS of 141.7 ± 2.2 nm, PDI of 0.278, ZP of 14.79 mV, %EE of 67.71%, and %LC of 37.34. ATR-IR analysis confirmed the compatibility between IMP and other formulation components, as well as the successful cross-linking reaction between CS and STPP and the entrapment of IMP within the nanoparticles. In vitro and ex vivo release studies of IMP-CS NPs revealed a biphasic release pattern characterized by an initial rapid burst effect followed by sustained release. The release mechanism was best described by the Higuchi model, followed by Fickian diffusion. Furthermore, formulation F4-50 was successfully incorporated into PF-ISG, demonstrating favorable attributes such as a clear physical appearance, pH resembling nasal mucosa (5.4), ideal gelation temperature of 33.6 °C (below nasal temperature), rapid gelation time (48.1 s), and adequate gel strength for nasal administration without causing local irritation. Moreover, the in vitro and ex vivo release profiles were enhanced with IMP-Cs NPs ISG, providing better control over the burst release effect of NPs. The optimized formulation exhibited stability over a one-month period, further validating its potential for therapeutic application.

## Figures and Tables

**Table 1:** Summary from the linear regression for the calibration of IMP at a range of 5–75 µg/mL.

Slope	Intercep	R^2^	LoD	LoQ
20.57	9.56	0.9999	3.71 µg/mL	11.25 µg/mL

**Table 2:** Characterization of Cs NPs formulations (Mean ± SD, *n* = 3).

Formulation Code	Cs (mg/mL)	STPP (mg/mL)	PS (nm)	PDI	ZP (mV)
F1	0.5	1	1294.9 ± 1.1	0.290 ± 0.20	7.320 ± 2.2
F2	1	1	1421.8 ± 12	0.332 ± 0.16	5.030 ± 1.4
F3	1.5	1	1149.5 ± 2.5	0.390 ± 0.090	9.110 ± 1.8
F4	2	1	180.60 ± 2.2	0.298 ± 0.070	15.47 ± 2.7
F5	2.5	1	310.60 ± 1.9	0.335 ± 0.080	14.78 ± 2.3
F6	3	1	266.20 ± 0.41	0.324 ± 0.040	10.83 ± 3.2
F7	1	0.5	128.70 ± 2.7	0.340 ± 0.010	16.38 ± 3.8

**Table 3:** Characterization of IMP-Cs NPs formulations (Mean ± SD, *n* = 3).

Formulation Code	PS (nm)	PDI	ZP (mV)	%EE	% LC
F4-10	132.9 ± 1.7	0.281 ± 0.020	18.48 ± 3.2	55.76 ± 0.26	23.28 ± 0.64
F4-50	141.7 ± 2.2	0.278 ± 0.080	16.79 ± 2.1	67.71 ± 1.9	37.34 ± 0.11
F7-10	130.0 ± 1.7	0.340 ± 0.060	15.78 ± 3.8	45.38 ± 1.6	8.380 ± 1.9
F7-50	134.2 ± 2.9	0.337 ± 0.040	15.71 ± 4.1	56.52 ± 1.4	11.02 ± 0.41

**Table 4:** Characterization of IMP Cs NPs ISG for gelation temperature, gelation time, viscosity, mucoadhesive strength, Gel capacity, and Gel strength (Mean ± SD, *n* = 3).

Formulation Code	T Sol-Gel (°C)	Gelation Time (s)	Viscosity (cps)	Mucoadhesive Strength (dyne/cm^2^)	Gel Strength (s)
F4-50-P1	33.6 ± 0.94	48.1 ± 0.70	1206 ± 1.6	990.3 ± 1.1	33 ± 1.2
F4-50-P2	30.3 ± 1.8	44.5 ± 0.60	1443 ± 1.1	1011 ± 0.46	41 ± 0.55

## Data Availability

The original contributions presented in the study are included in the article, further inquiries can be directed to the corresponding author (Samer Adwan).

## References

[1] Chen J., Finlay W.H., Vehring R., Martin A.R. (2024). Characterizing regional drug delivery within the nasal airways. Expert. Opin. Drug Deliv..

[2] Rai G., Gauba P., Dang S. (2023). Recent advances in nanotechnology for intra-nasal drug delivery and clinical applications. J. Drug Deliv. Sci. Technol..

[3] Jeong S.H., Jang J.H., Lee Y.B. (2023). Drug delivery to the brain via the nasal route of administration: Exploration of key targets and major consideration factors. J. Pharm. Investig..

[4] Huang Q., Chen X., Yu S., Gong G., Shu H. (2024). Research progress in brain-targeted nasal drug delivery. Front. Aging Neurosci..

[5] Wong C.Y.J., Baldelli A., Tietz O., van der Hoven J., Suman J., Ong H.X., Traini D. (2024). An overview of in vitro and in vivo techniques for characterization of intranasal protein and peptide formulations for brain targeting. Int. J. Pharm..

[6] Rutvik K., Meshva P., Dinal P., Mansi D. (2023). The nasal route, advanced drug delivery systems and evaluation: A review. Egypt. J. Chest Dis. Tuberc..

[7] Shaghlil L., Alshishani A., Sa’aleek A.A., Abdelkader H., Al-ebini Y. (2022). Formulation and evaluation of nasal insert for nose-to-brain drug delivery of rivastigmine tartrate. J. Drug Deliv. Sci. Technol..

[8] Kaur G., Goyal J., Behera P.K., Devi S., Singh S.K., Garg V., Mittal N. (2023). Unraveling the role of chitosan for nasal drug delivery systems: A review. Carbohydr. Polym. Technol. Appl..

[9] Aderibigbe B.A., Naki T. (2018). Design and efficacy of nanogels formulations for intranasal administration. Molecules.

[10] Saeed R.M., Dmour I., Taha M.O. (2020). Stable chitosan-based nanoparticles using polyphosphoric acid or hexametaphosphate for tandem ionotropic/covalent crosslinking and subsequent investigation as novel vehicles for drug delivery. Front. Bioeng. Biotechnol..

[11] Jha R., Mayanovic R.A. (2023). A review of the preparation, characterization, and applications of chitosan nanoparticles in nanomedicine. Nanomaterials.

[12] Awad R., Avital A., Sosnik A. (2023). Polymeric nanocarriers for nose-to-brain drug delivery in neurodegenerative diseases and neurodevelopmental disorders. Acta Pharm. Sin. B.

[13] Xu K., Duan S., Wang W., Ouyang Q., Qin F., Guo P., Qin M. (2024). Nose-to-brain delivery of nanotherapeutics: Transport mechanisms and applications. Wiley Interdiscip. Rev. Nanomed. Nanobiotechnol..

[14] Haider A., Khan S., Iqbal D.N., Shrahili M., Haider S., Mohammad K., Mustafa G. (2024). Advances in chitosan-based drug delivery systems: A comprehensive review for therapeutic applications. Eur. Polym. J..

[15] El-Araby A., Janati W., Ullah R., Ercisli S., Errachidi F. (2024). Chitosan, chitosan derivatives, and chitosan-based nanocomposites: Eco-friendly materials for advanced applications (a review). Front. Chem..

[16] Calvo P., Remunan-Lopez C., Vila-Jato J.L., Alonso M.J. (1997). Novel hydrophilic chitosan-polyethylene oxide nanoparticles as protein carriers. J. Appl. Polym. Sci..

[17] Mi Y., Chen Y., Gu G., Miao Q., Tan W., Li Q., Guo Z. (2021). New synthetic adriamycin-incorporated chitosan nanoparticles with enhanced antioxidant, antitumor activities and pH-sensitive drug release. Carbohydr. Polym..

[18] Jadhav A., Dharashive V., Shafi S., Chavan S., Honrao M., Inje R., Biradar A. (2024). A novel approach for nasal drug delivery system. Asian J. Pharm. Res. Dev..

[19] Shriky B., Vigato A.A., Sepulveda A.F., Machado I.P., de Araujo D.R. (2023). Poloxamer-based nanogels as delivery systems: How structural requirements can drive their biological performance. Biophys. Rev..

[20] Fayez R., Gupta V. (2024). Imipramine. StatPearls [Internet].

[21] Sipos B., Csóka I., Budai-Szűcs M., Kozma G., Berkesi D., Kónya Z., Katona G. (2021). Development of dexamethasone-loaded mixed polymeric micelles for nasal delivery. Eur. J. Pharm. Sci..

[22] Schmolka I.R. (1972). Artificial skin I. Preparation and properties of pluronic F-127 gels for treatment of burns. J. Biomed. Mater. Res..

[23] Kaur P., Garg T., Vaidya B., Prakash A., Rath G., Goyal A.K. (2015). Brain delivery of intranasal in situ gel of nanoparticulated polymeric carriers containing antidepressant drug: Behavioral and biochemical assessment. J. Drug Target..

[24] Radivojša M., Grabnar I., Grabnar P.A. (2013). Thermoreversible in situ gelling poloxamer-based systems with chitosan nanocomplexes for prolonged subcutaneous delivery of heparin: Design and in vitro evaluation. Eur. J. Pharm. Sci..

[25] Rarokar N.R., Saoji S.D., Raut N.A., Taksande J.B., Khedekar P.B., Dave V.S. (2016). Nanostructured cubosomes in a thermoresponsive depot system: An alternative approach for the controlled delivery of docetaxel. AAPS PharmSciTech.

[26] Rao M., Agrawal D.K., Shirsath C. (2017). Thermoreversible mucoadhesive in situ nasal gel for treatment of Parkinson’s disease. Drug Dev. Ind. Pharm..

[27] Alsarra I.A., Hamed A.Y., Mahrous G.M., El Maghraby G.M., Al-Robayan A.A., Alanazi F.K. (2009). Mucoadhesive polymeric hydrogels for nasal delivery of acyclovir. Drug Dev. Ind. Pharm..

[28] Madni A., Kashif P.M., Nazir I., Tahir N., Rehman M., Khan M.I., Jabar A. (2017). Drug-Polymer Interaction Studies of Cytarabine Loaded Chitosan Nanoparticles. J. Chem. Soc. Pak..

[29] Shoueir K.R., El-Desouky N., Rashad M.M., Ahmed M.K., Janowska I., El-Kemary M. (2021). Chitosan based-nanoparticles and nanocapsules: Overview, physicochemical features, applications of a nanofibrous scaffold, and bioprinting. Int. J. Biol. Macromol..

[30] Gomathi T., Sudha P.N., Florence J.A.K., Venkatesan J., Anil S. (2017). Fabrication of letrozole formulation using chitosan nanoparticles through ionic gelation method. Int. J. Biol. Macromol..

[31] Mistry A., Stolnik S., Illum L. (2009). Nanoparticles for direct nose-to-brain delivery of drugs. Int. J. Pharm..

[32] Md S., Khan R.A., Mustafa G., Chuttani K., Baboota S., Sahni J.K., Ali J. (2013). Bromocriptine loaded chitosan nanoparticles intended for direct nose to brain delivery: Pharmacodynamic, pharmacokinetic and scintigraphy study in mice model. Eur. J. Pharm. Sci..

[33] Badran M.M., Alanazi A.E., Ibrahim M.A., Alshora D.H., Taha E., Alomrani A.H. (2023). Optimization of bromocriptine-mesylate-loaded polycaprolactone nanoparticles coated with chitosan for nose-to-brain delivery: In vitro and in vivo studies. Polymers.

[34] Jingou J., Shilei H., Weiqi L., Danjun W., Tengfei W., Yi X. (2011). Preparation, characterization of hydrophilic and hydrophobic drug in combine loaded chitosan/cyclodextrin nanoparticles and in vitro release study. Colloids Surf. B Biointerfaces.

[35] Fan W., Yan W., Xu Z., Ni H. (2012). Formation mechanism of monodisperse, low molecular weight chitosan nanoparticles by ionic gelation technique. Colloids Surf. B Biointerfaces.

[36] Fernández-Urrusuno R., Calvo P., Remuñán-López C., Vila-Jato J.L., José Alonso M. (1999). Enhancement of nasal absorption of insulin using chitosan nanoparticles. Pharm. Res..

[37] Lazaridou M., Christodoulou E., Nerantzaki M., Kostoglou M., Lambropoulou D.A., Katsarou A., Bikiaris D.N. (2020). Formulation and in-vitro characterization of chitosan-nanoparticles loaded with the iron chelator deferoxamine mesylate (DFO). Pharmaceutics.

[38] Lakshmi P.K., Harini K. (2019). Design and optimization of thermo-reversible nasal in situ gel of atomoxetine hydrochloride using taguchi orthogonal array design. Dhaka Univ. J. Pharm. Sci..

[39] Abdel Bary G. (2014). Preparation and characterization of thermosensitive mucoadhesive in situ gels for nasal delivery of ondansetron hydrochloride. Al-Azhar J. Pharm. Sci..

[40] Beule A.G. (2010). Funktionen und Funktionsstörungen der respiratorischen Schleimhaut der Nase und der Nasennebenhöhlen. Laryngo-Rhino-Otologie.

[41] Badran M.M., Harisa G.I., AlQahtani S.A., Alanazi F.K., Zoheir K.M. (2016). Pravastatin-loaded chitosan nanoparticles: Formulation, characterization and cytotoxicity studies. J. Drug Deliv. Sci. Technol..

[42] Rub M.A., Azum N., Kumar D., Asiri A.M. (2022). Interaction of TX-100 and antidepressant imipramine hydrochloride drug mixture: Surface tension, 1H NMR, and FT-IR investigation. Gels.

[43] Wilson B., Alobaid B.N.M., Geetha K.M., Jenita J.L. (2021). Chitosan nanoparticles to enhance nasal absorption and brain targeting of sitagliptin to treat Alzheimer’s disease. J. Drug Deliv. Sci. Technol..

